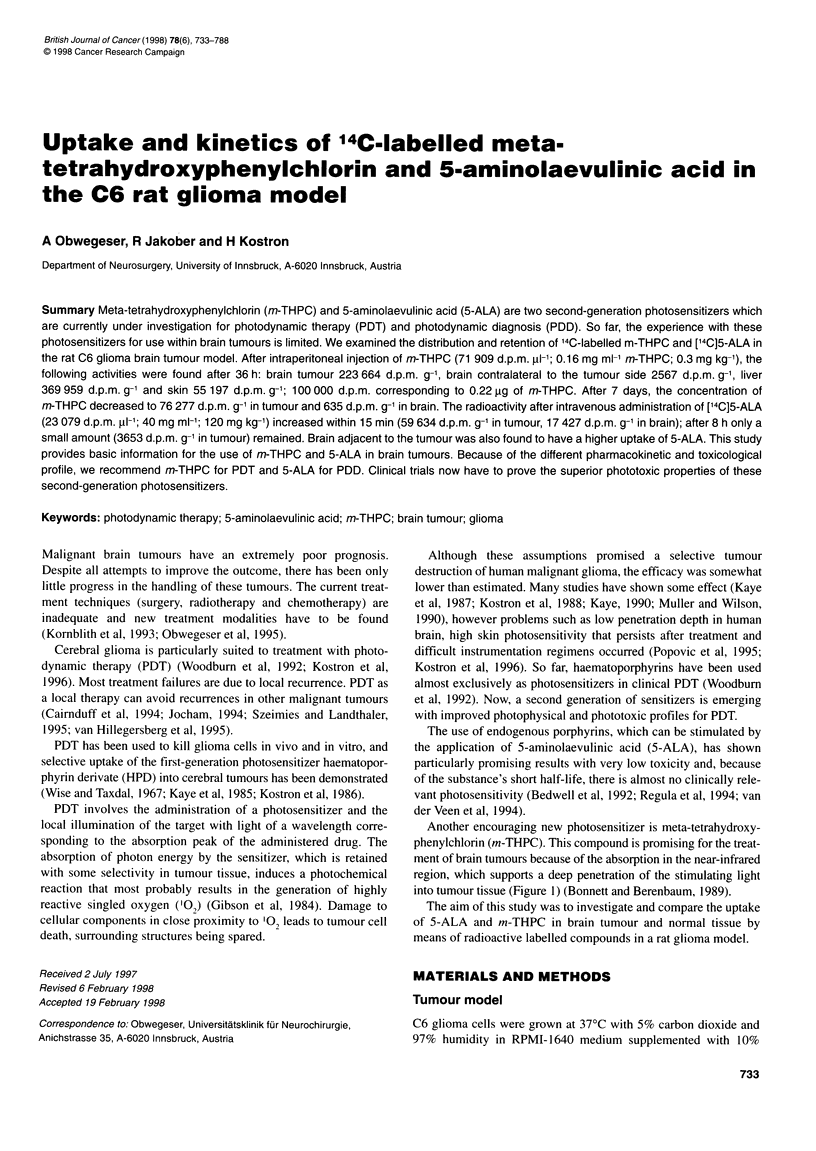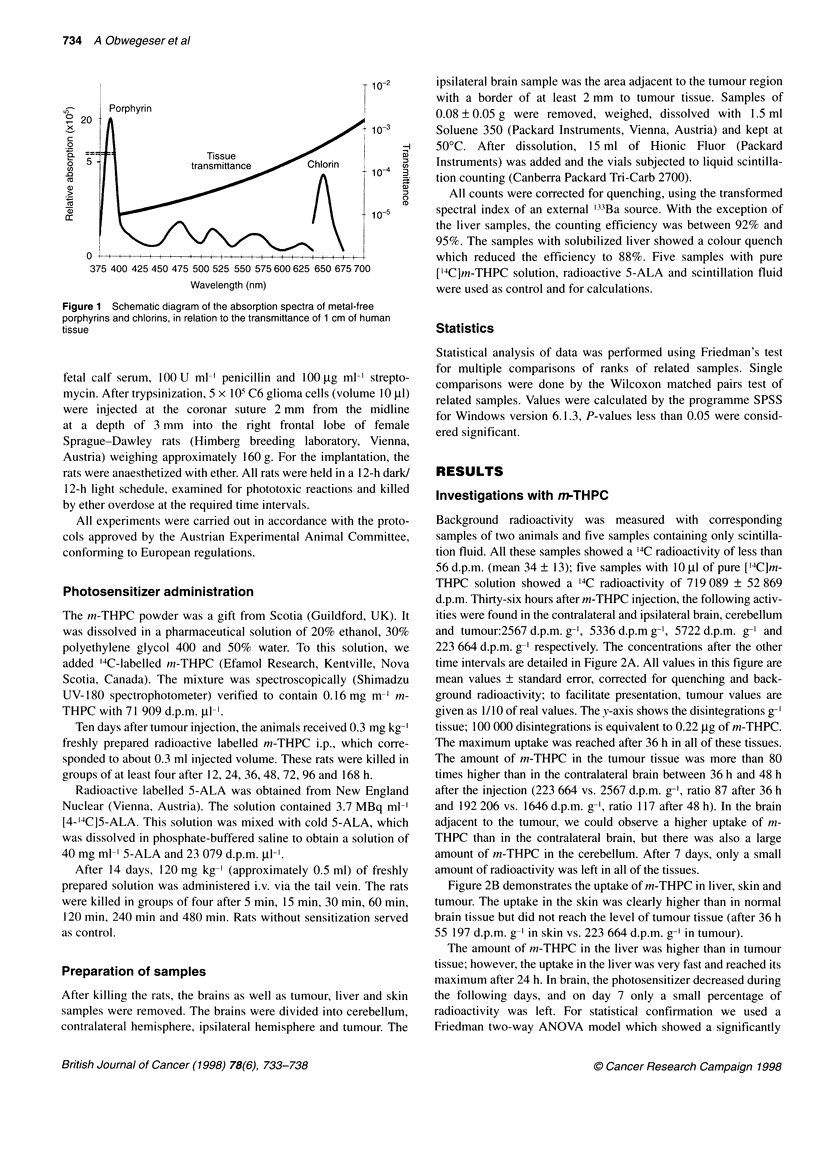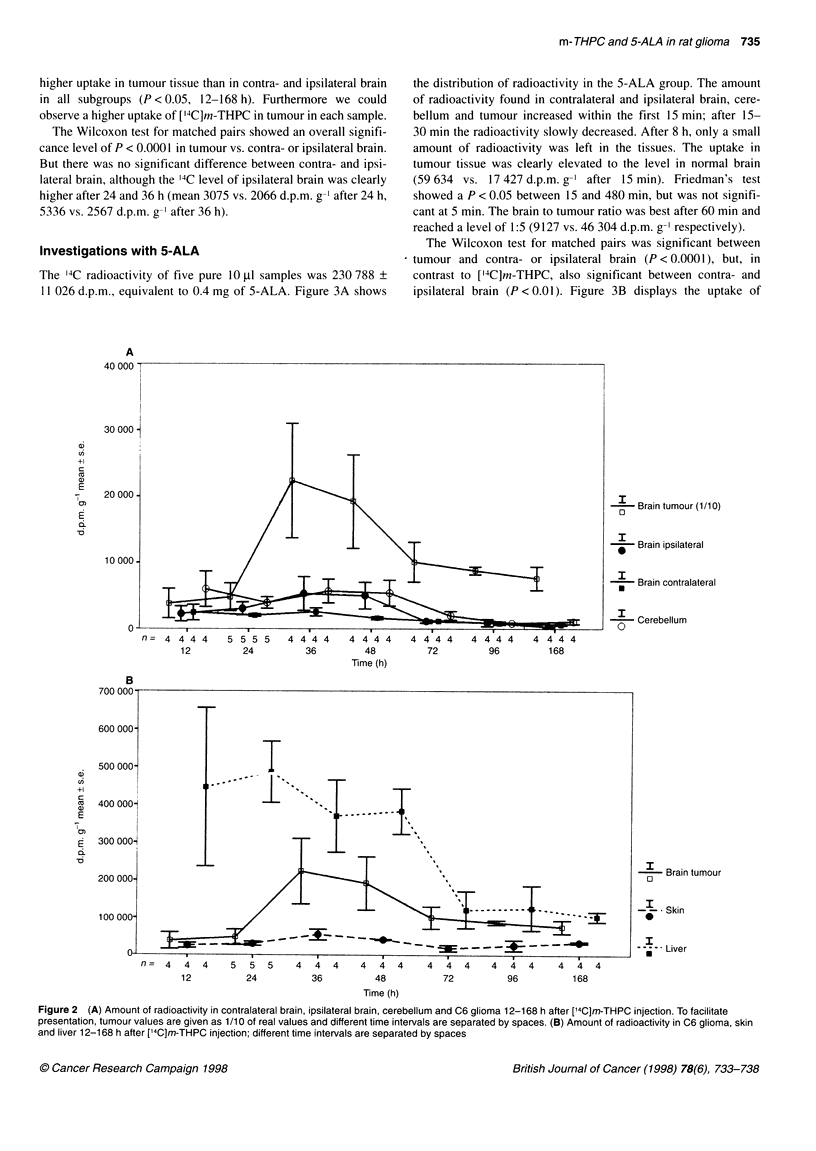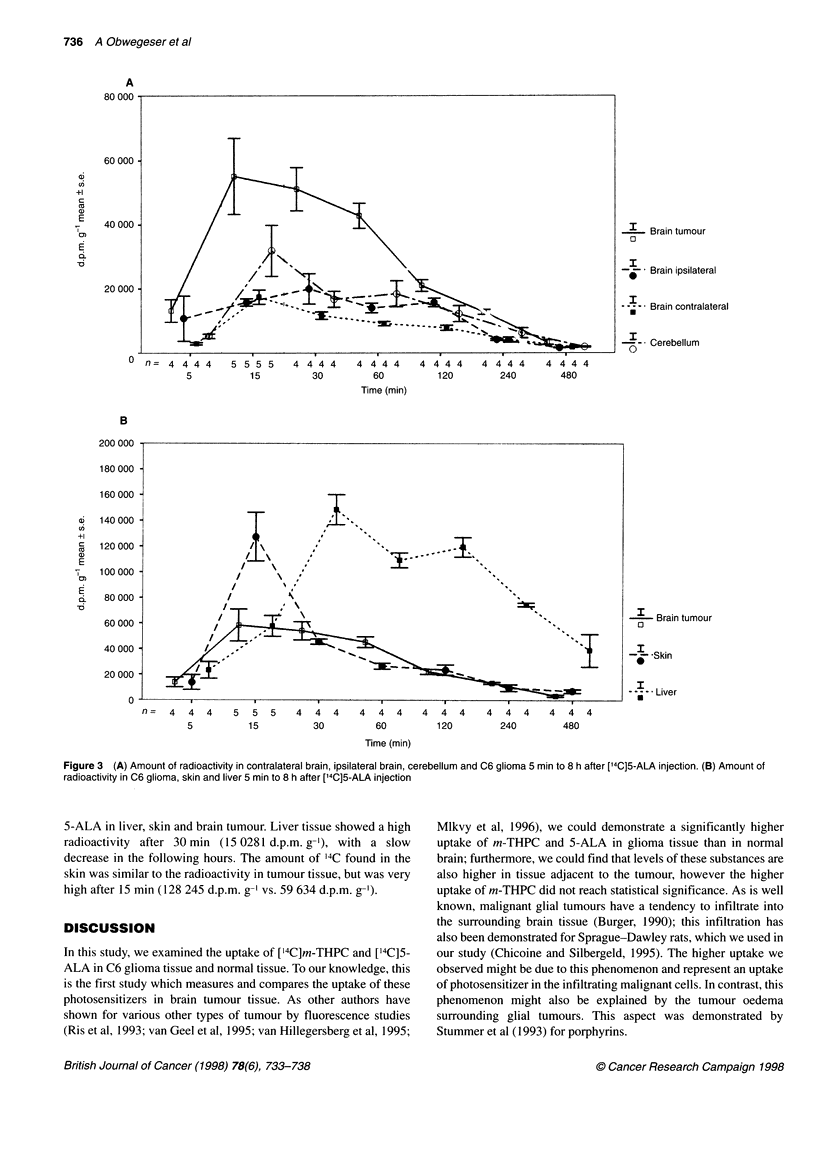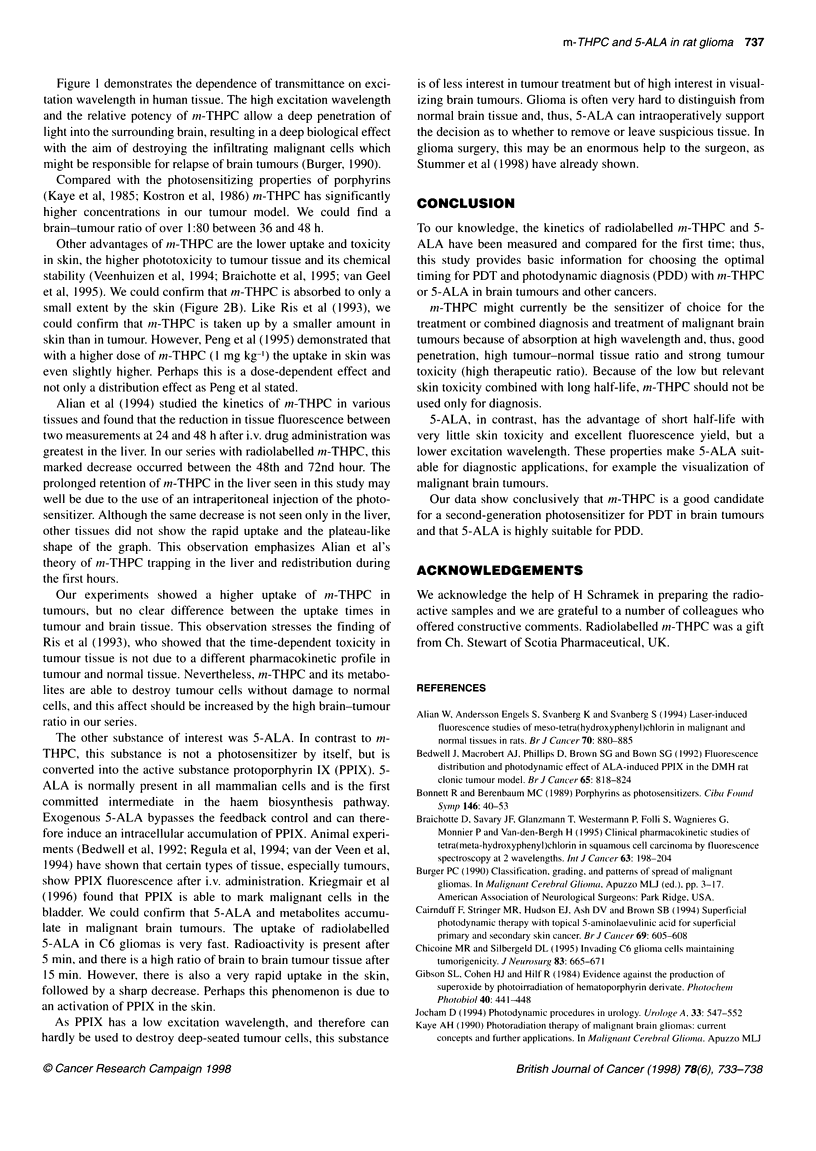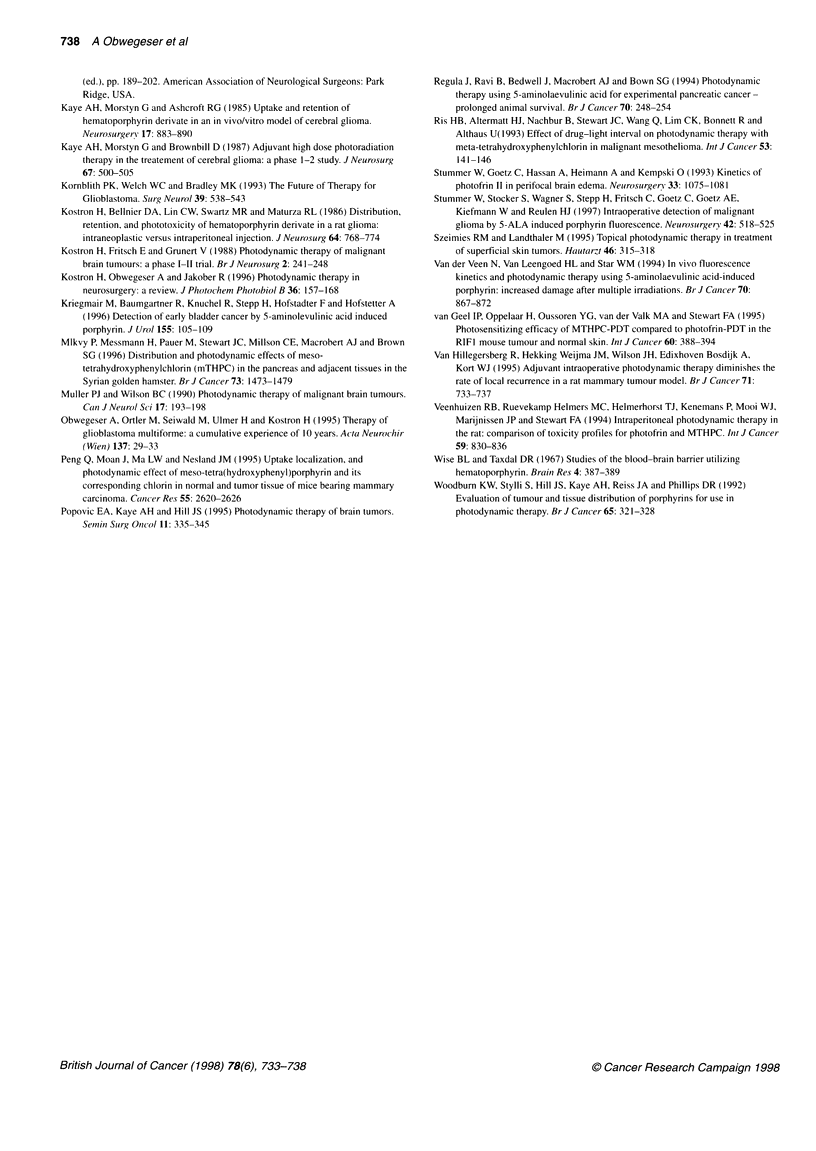# Uptake and kinetics of 14C-labelled meta-tetrahydroxyphenylchlorin and 5-aminolaevulinic acid in the C6 rat glioma model.

**DOI:** 10.1038/bjc.1998.569

**Published:** 1998-09

**Authors:** A. Obwegeser, R. Jakober, H. Kostron

**Affiliations:** Department of Neurosurgery, University of Innsbruck, Austria.

## Abstract

Meta-tetrahydroxyphenylchlorin (m-THPC) and 5-aminolaevulinic acid (5-ALA) are two second-generation photosensitizers which are currently under investigation for photodynamic therapy (PDT) and photodynamic diagnosis (PDD). So far, the experience with these photosensitizers for use within brain tumours is limited. We examined the distribution and retention of 14C-labelled m-THPC and [14C]5-ALA in the rat C6 glioma brain tumour model. After intraperitoneal injection of m-THPC (71,909 d.p.m. microl(-1); 0.16 mg ml(-1) m-THPC; 0.3 mg kg(-1)), the following activities were found after 36 h: brain tumour 223,664 d.p.m. g(-1), brain contralateral to the tumour side 2567 d.p.m. g(-1), liver 369,959 d.p.m. g(-1) and skin 55,197 d.p.m. g(-1); 100,000 d.p.m. corresponding to 0.22 microg of m-THPC. After 7 days, the concentration of m-THPC decreased to 76,277 d.p.m. g(-1) in tumour and 635 d.p.m. g(-1) in brain. The radioactivity after intravenous administration of [14C]5-ALA (23,079 d.p.m. microl(-1); 40 mg ml(-1); 120 mg kg(-1)) increased within 15 min (59,634 d.p.m. g(-1) in tumour, 17,427 d.p.m. g(-1) in brain); after 8 h only a small amount (3653 d.p.m. g(-1) in tumour) remained. Brain adjacent to the tumour was also found to have a higher uptake of 5-ALA. This study provides basic information for the use of m-THPC and 5-ALA in brain tumours. Because of the different pharmacokinetic and toxicological profile, we recommend m-THPC for PDT and 5-ALA for PDD. Clinical trials now have to prove the superior phototoxic properties of these second-generation photosensitizers.


British Journal of Cancer (1998) 78(6), 733–788
© 1998 Cancer Research Campaign

### Uptake and kinetics of $^{14}$C-labelled meta-tetrahydroxyphenylchlorin and 5-aminolaevulinic acid in the C6 rat glioma model

**A Obwegeser, R Jakober and H Kostron**

Department of Neurosurgery, University of Innsbruck, A-6020 Innsbruck, Austria

**Summary** Meta-tetrahydroxyphenylchlorin (*m*-THPC) and 5-aminolaevulinic acid (5-ALA) are two second-generation photosensitizers which are currently under investigation for photodynamic therapy (PDT) and photodynamic diagnosis (PDD). So far, the experience with these photosensitizers for use within brain tumours is limited. We examined the distribution and retention of $^{14}$C-labelled m-THPC and [$^{14}$C]5-ALA in the rat C6 glioma brain tumour model. After intraperitoneal injection of *m*-THPC (71 909 d.p.m. $\mu l^{-1}$; 0.16 mg $ml^{-1}$ *m*-THPC; 0.3 mg $kg^{-1}$), the following activities were found after 36 h: brain tumour 223 664 d.p.m. $g^{-1}$, brain contralateral to the tumour side 2567 d.p.m. $g^{-1}$, liver 369 959 d.p.m. $g^{-1}$ and skin 55 197 d.p.m. $g^{-1}$; 100 000 d.p.m. corresponding to 0.22 μg of *m*-THPC. After 7 days, the concentration of *m*-THPC decreased to 76 277 d.p.m. $g^{-1}$ in tumour and 635 d.p.m. $g^{-1}$ in brain. The radioactivity after intravenous administration of [$^{14}$C]5-ALA (23 079 d.p.m. $\mu l^{-1}$; 40 mg $ml^{-1}$; 120 mg $kg^{-1}$) increased within 15 min (59 634 d.p.m. $g^{-1}$ in tumour, 17 427 d.p.m. $g^{-1}$ in brain); after 8 h only a small amount (3653 d.p.m. $g^{-1}$ in tumour) remained. Brain adjacent to the tumour was also found to have a higher uptake of 5-ALA. This study provides basic information for the use of *m*-THPC and 5-ALA in brain tumours. Because of the different pharmacokinetic and toxicological profile, we recommend *m*-THPC for PDT and 5-ALA for PDD. Clinical trials now have to prove the superior phototoxic properties of these second-generation photosensitizers.

**Keywords:** photodynamic therapy; 5-aminolaevulinic acid; *m*-THPC; brain tumour; glioma

Malignant brain tumours have an extremely poor prognosis. Despite all attempts to improve the outcome, there has been only little progress in the handling of these tumours. The current treatment techniques (surgery, radiotherapy and chemotherapy) are inadequate and new treatment modalities have to be found (Kornblith et al, 1993; Obwegeser et al, 1995).

Cerebral glioma is particularly suited to treatment with photodynamic therapy (PDT) (Woodburn et al, 1992; Kostron et al, 1996). Most treatment failures are due to local recurrence. PDT as a local therapy can avoid recurrences in other malignant tumours (Cairnduff et al, 1994; Jocham, 1994; Szeimies and Landthaler, 1995; van Hillegersberg et al, 1995).

PDT has been used to kill glioma cells in vivo and in vitro, and selective uptake of the first-generation photosensitizer haematoporphyrin derivate (HPD) into cerebral tumours has been demonstrated (Wise and Taxdal, 1967; Kaye et al, 1985; Kostron et al, 1986).

PDT involves the administration of a photosensitizer and the local illumination of the target with light of a wavelength corresponding to the absorption peak of the administered drug. The absorption of photon energy by the sensitizer, which is retained with some selectivity in tumour tissue, induces a photochemical reaction that most probably results in the generation of highly reactive singled oxygen ($^1O_2$) (Gibson et al, 1984). Damage to cellular components in close proximity to $^1O_2$ leads to tumour cell death, surrounding structures being spared.

*Received 2 July 1997*
*Revised 6 February 1998*
*Accepted 19 February 1998*

*Correspondence to:* Obwegeser, Universitätsklinik für Neurochirurgie, Anichstrasse 35, A-6020 Innsbruck, Austria

Although these assumptions promised a selective tumour destruction of human malignant glioma, the efficacy was somewhat lower than estimated. Many studies have shown some effect (Kaye et al, 1987; Kostron et al, 1988; Kaye, 1990; Muller and Wilson, 1990), however problems such as low penetration depth in human brain, high skin photosensitivity that persists after treatment and difficult instrumentation regimens occurred (Popovic et al, 1995; Kostron et al, 1996). So far, haematoporphyrins have been used almost exclusively as photosensitizers in clinical PDT (Woodburn et al, 1992). Now, a second generation of sensitizers is emerging with improved photophysical and phototoxic profiles for PDT.

The use of endogenous porphyrins, which can be stimulated by the application of 5-aminolaevulinic acid (5-ALA), has shown particularly promising results with very low toxicity and, because of the substance's short half-life, there is almost no clinically relevant photosensitivity (Bedwell et al, 1992; Regula et al, 1994; van der Veen et al, 1994).

Another encouraging new photosensitizer is meta-tetrahydroxyphenylchlorin (*m*-THPC). This compound is promising for the treatment of brain tumours because of the absorption in the near-infrared region, which supports a deep penetration of the stimulating light into tumour tissue (Figure 1) (Bonnett and Berenbaum, 1989).

The aim of this study was to investigate and compare the uptake of 5-ALA and *m*-THPC in brain tumour and normal tissue by means of radioactive labelled compounds in a rat glioma model.

### MATERIALS AND METHODS

### Tumour model

C6 glioma cells were grown at 37°C with 5% carbon dioxide and 97% humidity in RPMI-1640 medium supplemented with 10%

**Figure 1** Schematic diagram of the absorption spectra of metal-free porphyrins and chlorins, in relation to the transmittance of 1 cm of human tissue fetal calf serum, 100 U $ml^{-1}$ penicillin and 100 μg $ml^{-1}$ streptomycin. After trypsinization, $5 \times 10^5$ C6 glioma cells (volume 10 μl) were injected at the coronar suture 2 mm from the midline at a depth of 3 mm into the right frontal lobe of female Sprague–Dawley rats (Himberg breeding laboratory, Vienna, Austria) weighing approximately 160 g. For the implantation, the rats were anaesthetized with ether. All rats were held in a 12-h dark/ 12-h light schedule, examined for phototoxic reactions and killed by ether overdose at the required time intervals.

All experiments were carried out in accordance with the protocols approved by the Austrian Experimental Animal Committee, conforming to European regulations.

### Photosensitizer administration

The *m*-THPC powder was a gift from Scotia (Guildford, UK). It was dissolved in a pharmaceutical solution of 20% ethanol, 30% polyethylene glycol 400 and 50% water. To this solution, we added $^{14}C$-labelled *m*-THPC (Efamol Research, Kentville, Nova Scotia, Canada). The mixture was spectroscopically (Shimadzu UV-180 spectrophotometer) verified to contain 0.16 mg $m^{-1}$ *m*-THPC with 71 909 d.p.m. $μl^{-1}$.

Ten days after tumour injection, the animals received 0.3 mg $kg^{-1}$ freshly prepared radioactive labelled *m*-THPC i.p., which corresponded to about 0.3 ml injected volume. These rats were killed in groups of at least four after 12, 24, 36, 48, 72, 96 and 168 h.

Radioactive labelled 5-ALA was obtained from New England Nuclear (Vienna, Austria). The solution contained 3.7 MBq $ml^{-1}$ [4-$^{14}C$]5-ALA. This solution was mixed with cold 5-ALA, which was dissolved in phosphate-buffered saline to obtain a solution of 40 mg $ml^{-1}$ 5-ALA and 23 079 d.p.m. $μl^{-1}$.

After 14 days, 120 mg $kg^{-1}$ (approximately 0.5 ml) of freshly prepared solution was administered i.v. via the tail vein. The rats were killed in groups of four after 5 min, 15 min, 30 min, 60 min, 120 min, 240 min and 480 min. Rats without sensitization served as control.

### Preparation of samples

After killing the rats, the brains as well as tumour, liver and skin samples were removed. The brains were divided into cerebellum, contralateral hemisphere, ipsilateral hemisphere and tumour. The ipsilateral brain sample was the area adjacent to the tumour region with a border of at least 2 mm to tumour tissue. Samples of 0.08 ± 0.05 g were removed, weighed, dissolved with 1.5 ml Soluene 350 (Packard Instruments, Vienna, Austria) and kept at 50°C. After dissolution, 15 ml of Hionic Fluor (Packard Instruments) was added and the vials subjected to liquid scintillation counting (Canberra Packard Tri-Carb 2700).

All counts were corrected for quenching, using the transformed spectral index of an external $^{133}Ba$ source. With the exception of the liver samples, the counting efficiency was between 92% and 95%. The samples with solubilized liver showed a colour quench which reduced the efficiency to 88%. Five samples with pure [$^{14}C$]*m*-THPC solution, radioactive 5-ALA and scintillation fluid were used as control and for calculations.

### Statistics

Statistical analysis of data was performed using Friedman's test for multiple comparisons of ranks of related samples. Single comparisons were done by the Wilcoxon matched pairs test of related samples. Values were calculated by the programme SPSS for Windows version 6.1.3, *P*-values less than 0.05 were considered significant.

### RESULTS

### Investigations with *m*-THPC

Background radioactivity was measured with corresponding samples of two animals and five samples containing only scintillation fluid. All these samples showed a $^{14}C$ radioactivity of less than 56 d.p.m. (mean 34 ± 13); five samples with 10 μl of pure [$^{14}C$]*m*-THPC solution showed a $^{14}C$ radioactivity of 719 089 ± 52 869 d.p.m. Thirty-six hours after *m*-THPC injection, the following activities were found in the contralateral and ipsilateral brain, cerebellum and tumour:2567 d.p.m. $g^{-1}$, 5336 d.p.m $g^{-1}$, 5722 d.p.m. $g^{-1}$ and 223 664 d.p.m. $g^{-1}$ respectively. The concentrations after the other time intervals are detailed in Figure 2A. All values in this figure are mean values ± standard error, corrected for quenching and background radioactivity; to facilitate presentation, tumour values are given as 1/10 of real values. The *y*-axis shows the disintegrations $g^{-1}$ tissue; 100 000 disintegrations is equivalent to 0.22 μg of *m*-THPC. The maximum uptake was reached after 36 h in all of these tissues. The amount of *m*-THPC in the tumour tissue was more than 80 times higher than in the contralateral brain between 36 h and 48 h after the injection (223 664 vs. 2567 d.p.m. $g^{-1}$, ratio 87 after 36 h and 192 206 vs. 1646 d.p.m. $g^{-1}$, ratio 117 after 48 h). In the brain adjacent to the tumour, we could observe a higher uptake of *m*-THPC than in the contralateral brain, but there was also a large amount of *m*-THPC in the cerebellum. After 7 days, only a small amount of radioactivity was left in all of the tissues.

Figure 2B demonstrates the uptake of *m*-THPC in liver, skin and tumour. The uptake in the skin was clearly higher than in normal brain tissue but did not reach the level of tumour tissue (after 36 h 55 197 d.p.m. $g^{-1}$ in skin vs. 223 664 d.p.m. $g^{-1}$ in tumour).

The amount of *m*-THPC in the liver was higher than in tumour tissue; however, the uptake in the liver was very fast and reached its maximum after 24 h. In brain, the photosensitizer decreased during the following days, and on day 7 only a small percentage of radioactivity was left. For statistical confirmation we used a Friedman two-way ANOVA model which showed a significantly

© Cancer Research Campaign 1998

higher uptake in tumour tissue than in contra- and ipsilateral brain in all subgroups ($P < 0.05$, 12–168 h). Furthermore we could observe a higher uptake of $[^{14}C]$*m*-THPC in tumour in each sample.

The Wilcoxon test for matched pairs showed an overall significance level of $P < 0.0001$ in tumour vs. contra- or ipsilateral brain. But there was no significant difference between contra- and ipsilateral brain, although the $^{14}C$ level of ipsilateral brain was clearly higher after 24 and 36 h (mean 3075 vs. 2066 d.p.m. $g^{-1}$ after 24 h, 5336 vs. 2567 d.p.m. $g^{-1}$ after 36 h).

### Investigations with 5-ALA

The $^{14}C$ radioactivity of five pure 10 µl samples was 230 788 ± 11 026 d.p.m., equivalent to 0.4 mg of 5-ALA. Figure 3A shows the distribution of radioactivity in the 5-ALA group. The amount of radioactivity found in contralateral and ipsilateral brain, cerebellum and tumour increased within the first 15 min; after 15–30 min the radioactivity slowly decreased. After 8 h, only a small amount of radioactivity was left in the tissues. The uptake in tumour tissue was clearly elevated to the level in normal brain (59 634 vs. 17 427 d.p.m. $g^{-1}$ after 15 min). Friedman's test showed a $P < 0.05$ between 15 and 480 min, but was not significant at 5 min. The brain to tumour ratio was best after 60 min and reached a level of 1:5 (9127 vs. 46 304 d.p.m. $g^{-1}$ respectively).

The Wilcoxon test for matched pairs was significant between tumour and contra- or ipsilateral brain ($P < 0.0001$), but, in contrast to $[^{14}C]$*m*-THPC, also significant between contra- and ipsilateral brain ($P < 0.01$). Figure 3B displays the uptake of

**Figure 2** (**A**) Amount of radioactivity in contralateral brain, ipsilateral brain, cerebellum and C6 glioma 12–168 h after $[^{14}C]$*m*-THPC injection. To facilitate presentation, tumour values are given as 1/10 of real values and different time intervals are separated by spaces. (**B**) Amount of radioactivity in C6 glioma, skin and liver 12–168 h after $[^{14}C]$*m*-THPC injection; different time intervals are separated by spaces

© *Cancer Research Campaign 1998*

**Figure 3** (**A**) Amount of radioactivity in contralateral brain, ipsilateral brain, cerebellum and C6 glioma 5 min to 8 h after [$^{14}$C]5-ALA injection. (**B**) Amount of radioactivity in C6 glioma, skin and liver 5 min to 8 h after [$^{14}$C]5-ALA injection

5-ALA in liver, skin and brain tumour. Liver tissue showed a high radioactivity after 30 min (15 0281 d.p.m. g$^{-1}$), with a slow decrease in the following hours. The amount of $^{14}$C found in the skin was similar to the radioactivity in tumour tissue, but was very high after 15 min (128 245 d.p.m. g$^{-1}$ vs. 59 634 d.p.m. g$^{-1}$).

### DISCUSSION

In this study, we examined the uptake of [$^{14}$C]*m*-THPC and [$^{14}$C]5-ALA in C6 glioma tissue and normal tissue. To our knowledge, this is the first study which measures and compares the uptake of these photosensitizers in brain tumour tissue. As other authors have shown for various other types of tumour by fluorescence studies (Ris et al, 1993; van Geel et al, 1995; van Hillegersberg et al, 1995; Mlkvy et al, 1996), we could demonstrate a significantly higher uptake of *m*-THPC and 5-ALA in glioma tissue than in normal brain; furthermore, we could find that levels of these substances are also higher in tissue adjacent to the tumour, however the higher uptake of *m*-THPC did not reach statistical significance. As is well known, malignant glial tumours have a tendency to infiltrate into the surrounding brain tissue (Burger, 1990); this infiltration has also been demonstrated for Sprague–Dawley rats, which we used in our study (Chicoine and Silbergeld, 1995). The higher uptake we observed might be due to this phenomenon and represent an uptake of photosensitizer in the infiltrating malignant cells. In contrast, this phenomenon might also be explained by the tumour oedema surrounding glial tumours. This aspect was demonstrated by Stummer et al (1993) for porphyrins.

© Cancer Research Campaign 1998

Figure 1 demonstrates the dependence of transmittance on excitation wavelength in human tissue. The high excitation wavelength and the relative potency of *m*-THPC allow a deep penetration of light into the surrounding brain, resulting in a deep biological effect with the aim of destroying the infiltrating malignant cells which might be responsible for relapse of brain tumours (Burger, 1990).

Compared with the photosensitizing properties of porphyrins (Kaye et al, 1985; Kostron et al, 1986) *m*-THPC has significantly higher concentrations in our tumour model. We could find a brain–tumour ratio of over 1:80 between 36 and 48 h.

Other advantages of *m*-THPC are the lower uptake and toxicity in skin, the higher phototoxicity to tumour tissue and its chemical stability (Veenhuizen et al, 1994; Braichotte et al, 1995; van Geel et al, 1995). We could confirm that *m*-THPC is absorbed to only a small extent by the skin (Figure 2B). Like Ris et al (1993), we could confirm that *m*-THPC is taken up by a smaller amount in skin than in tumour. However, Peng et al (1995) demonstrated that with a higher dose of *m*-THPC (1 mg $kg^{-1}$) the uptake in skin was even slightly higher. Perhaps this is a dose-dependent effect and not only a distribution effect as Peng et al stated.

Alian et al (1994) studied the kinetics of *m*-THPC in various tissues and found that the reduction in tissue fluorescence between two measurements at 24 and 48 h after i.v. drug administration was greatest in the liver. In our series with radiolabelled *m*-THPC, this marked decrease occurred between the 48th and 72nd hour. The prolonged retention of *m*-THPC in the liver seen in this study may well be due to the use of an intraperitoneal injection of the photosensitizer. Although the same decrease is not seen only in the liver, other tissues did not show the rapid uptake and the plateau-like shape of the graph. This observation emphasizes Alian et al's theory of *m*-THPC trapping in the liver and redistribution during the first hours.

Our experiments showed a higher uptake of *m*-THPC in tumours, but no clear difference between the uptake times in tumour and brain tissue. This observation stresses the finding of Ris et al (1993), who showed that the time-dependent toxicity in tumour tissue is not due to a different pharmacokinetic profile in tumour and normal tissue. Nevertheless, *m*-THPC and its metabolites are able to destroy tumour cells without damage to normal cells, and this affect should be increased by the high brain–tumour ratio in our series.

The other substance of interest was 5-ALA. In contrast to *m*-THPC, this substance is not a photosensitizer by itself, but is converted into the active substance protoporphyrin IX (PPIX). 5-ALA is normally present in all mammalian cells and is the first committed intermediate in the haem biosynthesis pathway. Exogenous 5-ALA bypasses the feedback control and can therefore induce an intracellular accumulation of PPIX. Animal experiments (Bedwell et al, 1992; Regula et al, 1994; van der Veen et al, 1994) have shown that certain types of tissue, especially tumours, show PPIX fluorescence after i.v. administration. Kriegmair et al (1996) found that PPIX is able to mark malignant cells in the bladder. We could confirm that 5-ALA and metabolites accumulate in malignant brain tumours. The uptake of radiolabelled 5-ALA in C6 gliomas is very fast. Radioactivity is present after 5 min, and there is a high ratio of brain to brain tumour tissue after 15 min. However, there is also a very rapid uptake in the skin, followed by a sharp decrease. Perhaps this phenomenon is due to an activation of PPIX in the skin.

As PPIX has a low excitation wavelength, and therefore can hardly be used to destroy deep-seated tumour cells, this substance is of less interest in tumour treatment but of high interest in visualizing brain tumours. Glioma is often very hard to distinguish from normal brain tissue and, thus, 5-ALA can intraoperatively support the decision as to whether to remove or leave suspicious tissue. In glioma surgery, this may be an enormous help to the surgeon, as Stummer et al (1998) have already shown.

### CONCLUSION

To our knowledge, the kinetics of radiolabelled *m*-THPC and 5-ALA have been measured and compared for the first time; thus, this study provides basic information for choosing the optimal timing for PDT and photodynamic diagnosis (PDD) with *m*-THPC or 5-ALA in brain tumours and other cancers.

*m*-THPC might currently be the sensitizer of choice for the treatment or combined diagnosis and treatment of malignant brain tumours because of absorption at high wavelength and, thus, good penetration, high tumour–normal tissue ratio and strong tumour toxicity (high therapeutic ratio). Because of the low but relevant skin toxicity combined with long half-life, *m*-THPC should not be used only for diagnosis.

5-ALA, in contrast, has the advantage of short half-life with very little skin toxicity and excellent fluorescence yield, but a lower excitation wavelength. These properties make 5-ALA suitable for diagnostic applications, for example the visualization of malignant brain tumours.

Our data show conclusively that *m*-THPC is a good candidate for a second-generation photosensitizer for PDT in brain tumours and that 5-ALA is highly suitable for PDD.

### ACKNOWLEDGEMENTS

We acknowledge the help of H Schramek in preparing the radioactive samples and we are grateful to a number of colleagues who offered constructive comments. Radiolabelled *m*-THPC was a gift from Ch. Stewart of Scotia Pharmaceutical, UK.

### REFERENCES

Alian W, Andersson Engels S, Svanberg K and Svanberg S (1994) Laser-induced fluorescence studies of meso-tetra(hydroxyphenyl)chlorin in malignant and normal tissues in rats. *Br J Cancer* **70**: 880–885

Bedwell J, Macrobert AJ, Phillips D, Brown SG and Bown SG (1992) Fluorescence distribution and photodynamic effect of ALA-induced PPIX in the DMH rat clonic tumour model. *Br J Cancer* **65**: 818–824

Bonnett R and Berenbaum MC (1989) Porphyrins as photosensitizers. *Ciba Found Symp* **146**: 40–53

Braichotte D, Savary JF, Glanzmann T, Westermann P, Folli S, Wagnieres G, Monnier P and Van-den-Bergh H (1995) Clinical pharmacokinetic studies of tetra(meta-hydroxyphenyl)chlorin in squamous cell carcinoma by fluorescence spectroscopy at 2 wavelengths. *Int J Cancer* **63**: 198–204

Burger PC (1990) Classification, grading, and patterns of spread of malignant gliomas. In *Malignant Cerebral Glioma*, Apuzzo MLJ (ed.), pp. 3–17. American Association of Neurological Surgeons: Park Ridge, USA.

Cairnduff F, Stringer MR, Hudson EJ, Ash DV and Brown SB (1994) Superficial photodynamic therapy with topical 5-aminolaevulinic acid for superficial primary and secondary skin cancer. *Br J Cancer* **69**: 605–608

Chicoine MR and Silbergeld DL (1995) Invading C6 glioma cells maintaining tumorigenicity. *J Neurosurg* **83**: 665–671

Gibson SL, Cohen HJ and Hilf R (1984) Evidence against the production of superoxide by photoirradiation of hematoporphyrin derivate. *Photochem Photobiol* **40**: 441–448

Jocham D (1994) Photodynamic procedures in urology. *Urologe A*, **33**: 547–552

Kaye AH (1990) Photoradiation therapy of malignant brain gliomas: current concepts and further applications. In *Malignant Cerebral Glioma*. Apuzzo MLJ

© Cancer Research Campaign 1998 

(ed.), pp. 189–202. American Association of Neurological Surgeons: Park Ridge, USA.

Kaye AH, Morstyn G and Ashcroft RG (1985) Uptake and retention of hematoporphyrin derivate in an in vivo/vitro model of cerebral glioma. *Neurosurgery* **17**: 883–890

Kaye AH, Morstyn G and Brownbill D (1987) Adjuvant high dose photoradiation therapy in the treatement of cerebral glioma: a phase 1–2 study. *J Neurosurg* **67**: 500–505

Kornblith PK, Welch WC and Bradley MK (1993) The Future of Therapy for Glioblastoma. *Surg Neurol* **39**: 538–543

Kostron H, Bellnier DA, Lin CW, Swartz MR and Maturza RL (1986) Distribution, retention, and phototoxicity of hematoporphyrin derivate in a rat glioma: intraneoplastic versus intraperitoneal injection. *J Neurosurg* **64**: 768–774

Kostron H, Fritsch E and Grunert V (1988) Photodynamic therapy of malignant brain tumours: a phase I–II trial. *Br J Neurosurg* **2**: 241–248

Kostron H, Obwegeser A and Jakober R (1996) Photodynamic therapy in neurosurgery: a review. *J Photochem Photobiol B* **36**: 157–168

Kriegmair M, Baumgartner R, Knuchel R, Stepp H, Hofstadter F and Hofstetter A (1996) Detection of early bladder cancer by 5-aminolevulinic acid induced porphyrin. *J Urol* **155**: 105–109

Mlkvy P, Messmann H, Pauer M, Stewart JC, Millson CE, Macrobert AJ and Brown SG (1996) Distribution and photodynamic effects of meso-tetrahydroxyphenylchlorin (mTHPC) in the pancreas and adjacent tissues in the Syrian golden hamster. *Br J Cancer* **73**: 1473–1479

Muller PJ and Wilson BC (1990) Photodynamic therapy of malignant brain tumours. *Can J Neurol Sci* **17**: 193–198

Obwegeser A, Ortler M, Seiwald M, Ulmer H and Kostron H (1995) Therapy of glioblastoma multiforme: a cumulative experience of 10 years. *Acta Neurochir (Wien)* **137**: 29–33

Peng Q, Moan J, Ma LW and Nesland JM (1995) Uptake localization, and photodynamic effect of meso-tetra(hydroxyphenyl)porphyrin and its corresponding chlorin in normal and tumor tissue of mice bearing mammary carcinoma. *Cancer Res* **55**: 2620–2626

Popovic EA, Kaye AH and Hill JS (1995) Photodynamic therapy of brain tumors. *Semin Surg Oncol* **11**: 335–345

Regula J, Ravi B, Bedwell J, Macrobert AJ and Bown SG (1994) Photodynamic therapy using 5-aminolaevulinic acid for experimental pancreatic cancer – prolonged animal survival. *Br J Cancer* **70**: 248–254

Ris HB, Altermatt HJ, Nachbur B, Stewart JC, Wang Q, Lim CK, Bonnett R and Althaus U(1993) Effect of drug–light interval on photodynamic therapy with meta-tetrahydroxyphenylchlorin in malignant mesothelioma. *Int J Cancer* **53**: 141–146

Stummer W, Goetz C, Hassan A, Heimann A and Kempski O (1993) Kinetics of photofrin II in perifocal brain edema. *Neurosurgery* **33**: 1075–1081

Stummer W, Stocker S, Wagner S, Stepp H, Fritsch C, Goetz C, Goetz AE, Kiefmann W and Reulen HJ (1997) Intraoperative detection of malignant glioma by 5-ALA induced porphyrin fluorescence. *Neurosurgery* **42**: 518–525

Szeimies RM and Landthaler M (1995) Topical photodynamic therapy in treatment of superficial skin tumors. *Hautarzt* **46**: 315–318

Van der Veen N, Van Leengoed HL and Star WM (1994) In vivo fluorescence kinetics and photodynamic therapy using 5-aminolaevulinic acid-induced porphyrin: increased damage after multiple irradiations. *Br J Cancer* **70**: 867–872

van Geel IP, Oppelaar H, Oussoren YG, van der Valk MA and Stewart FA (1995) Photosensitizing efficacy of MTHPC-PDT compared to photofrin-PDT in the RIF1 mouse tumour and normal skin. *Int J Cancer* **60**: 388–394

Van Hillegersberg R, Hekking Weijma JM, Wilson JH, Edixhoven Bosdijk A, Kort WJ (1995) Adjuvant intraoperative photodynamic therapy diminishes the rate of local recurrence in a rat mammary tumour model. *Br J Cancer* **71**: 733–737

Veenhuizen RB, Ruevekamp Helmers MC, Helmerhorst TJ, Kenemans P, Mooi WJ, Marijnissen JP and Stewart FA (1994) Intraperitoneal photodynamic therapy in the rat: comparison of toxicity profiles for photofrin and MTHPC. *Int J Cancer* **59**: 830–836

Wise BL and Taxdal DR (1967) Studies of the blood–brain barrier utilizing hematoporphyrin. *Brain Res* **4**: 387–389

Woodburn KW, Stylli S, Hill JS, Kaye AH, Reiss JA and Phillips DR (1992) Evaluation of tumour and tissue distribution of porphyrins for use in photodynamic therapy. *Br J Cancer* **65**: 321–328

 © Cancer Research Campaign 1998